# Supplementary material for: Exploring Conditions for Handling Packing and Shipping Aedes aegypti Males to Support an SIT Field Project in Brazil
Source: Insects. 2022 Sep 25;13(10):871. doi: 10.3390/insects13100871 (PMC9604236; doi:10.3390/insects13100871)
Supplement: Supplementary file 1 [file insects-13-00871-s001.zip › statistical_report.pdf]

Statistical Report

Impact of chilling

This section is dedicated to the first part of the manuscript to define the conditions to initiate the second part of handling, packing, and shipping.

Survival

The survival models for the temperatures and time intervals for knockout are presented below.

Time interval

Survival analysis of holding mosquitoes at 4 °C for different time intervals.

```
## Call:
## survdiff(formula = Surv(time, status) ~ treatment, data = handling_data2)
##
##                       N Observed Expected (O-E)^2/E (O-E)^2/V
## treatment=120     350       89     71.6      4.21      5.65
## treatment=60      350       82     72.5      1.24      1.67
## treatment=90      350       64     74.9      1.59      2.17
## treatment=Control 350       58     73.9      3.43      4.66
##
##  Chisq= 10.6  on 3 degrees of freedom, p= 0.01
```

Temperature

Survival analysis of holding mosquitoes for 60 minutes in different temperatures.

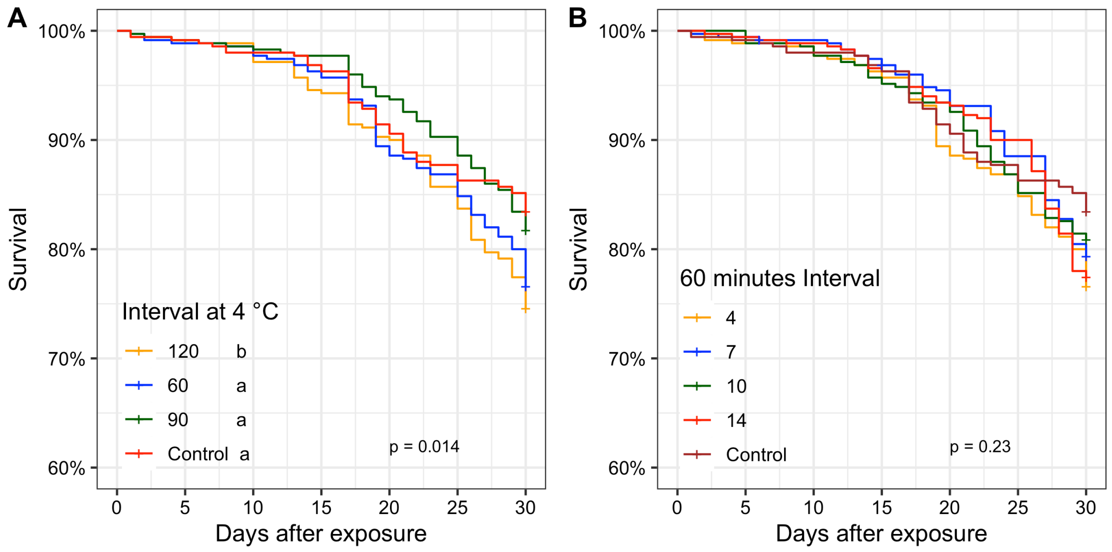

Figure 1. Chilling and time interval survival curve

Male escaping rate

Escaping rate analysis of males submitted to knockout/chilled conditions at different hours after emergence.

```
## # A tibble: 6 × 4
## # Groups:   compaction_interval, age_at_flight_test [2]
##   compaction_interval age_at_flight_test treatment  mean
##   <ord>               <ord>              <ord>     <dbl>
## 1 6hrs                72hrs              control   0.743
## 2 6hrs                72hrs              100       0.735
## 3 6hrs                72hrs              150       0.776
## 4 24hrs               96hrs              control   0.827
## 5 24hrs               96hrs              100       0.625
## 6 24hrs               96hrs              150       0.757

##
##  Kruskal-Wallis rank sum test
##
## data:  escaping_rate by interaction
## Kruskal-Wallis chi-squared = 8.3719, df = 3, p-value = 0.03892
```

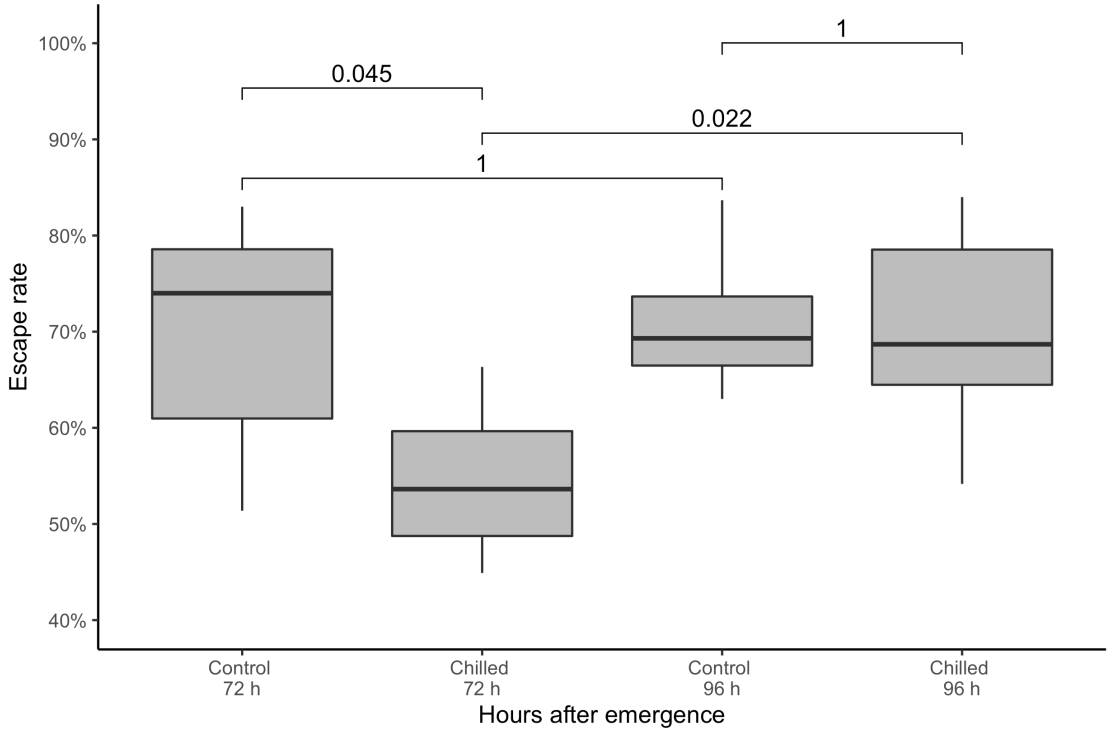

Figure 2. Male escaping rate between males of different ages (72 and 96 h post emergence) during chilled/knockout condition

Impact of packing and shipping

Survival

Compaction

Survival analysis comparing the two compaction levels at 100 and 150 males/cm^3.

```
## Call:
## survdiff(formula = Surv(time, status) ~ treatment + transport_time,
##     data = transport_data %>% filter(treatment == "100"))
##
##                                      N Observed Expected (O-E)^2/E (O-E)^2/V
## treatment=100, transport_time=6  600      209      225      1.18      2.48
## treatment=100, transport_time=24 600      233      217      1.23      2.48
##
##  Chisq= 2.5  on 1 degrees of freedom, p= 0.1
```

```
## Call:
## survdiff(formula = Surv(time, status) ~ treatment + transport_time,
##     data = transport_data %>% filter(treatment == "150"))
##
##                                      N Observed Expected (O-E)^2/E (O-E)^2/V
## treatment=150, transport_time=6  600      244      281      4.78      10.2
## treatment=150, transport_time=24 600      299      262      5.12      10.2
```

```
##
##  Chisq= 10.2  on 1 degrees of freedom, p= 0.001
```

Transportation time simulation

Survival analysis comparing the two transportation time simulation - 6 and 24 hours.

```
## Call:
## survdiff(formula = Surv(time, status) ~ treatment + transport_time,
##     data = transport_data %>% filter(transport_time %in% c(0,
##         6), treatment != "50"))
##
##                                           N Observed Expected (O-E)^2/E (O-E)
^2/V
## treatment=100, transport_time=6      600      209      160      14.7
19.8
## treatment=150, transport_time=6      600      244      151      57.1
75.6
## treatment=Chilled , transport_time=0 600      114      170      18.5
25.4
## treatment=Control, transport_time=0  600       89      174      41.8
58.1
##
##  Chisq= 135  on 3 degrees of freedom, p= <2e-16
```

```
## Call:
## survdiff(formula = Surv(time, status) ~ treatment + transport_time,
##     data = transport_data %>% filter(transport_time %in% c(0,
##         24), treatment != "50"))
##
##                                            N Observed Expected (O-E)^2/E (O-E
)^2/V
## treatment=100, transport_time=24      600      233      178      17.2
23.1
## treatment=150, transport_time=24      600      299      163     114.2
149.8
## treatment=Chilled , transport_time=0  600      114      195      33.5
46.5
## treatment=Control, transport_time=0   600       89      200      61.4
86.1
##
##  Chisq= 232  on 3 degrees of freedom, p= <2e-16
```

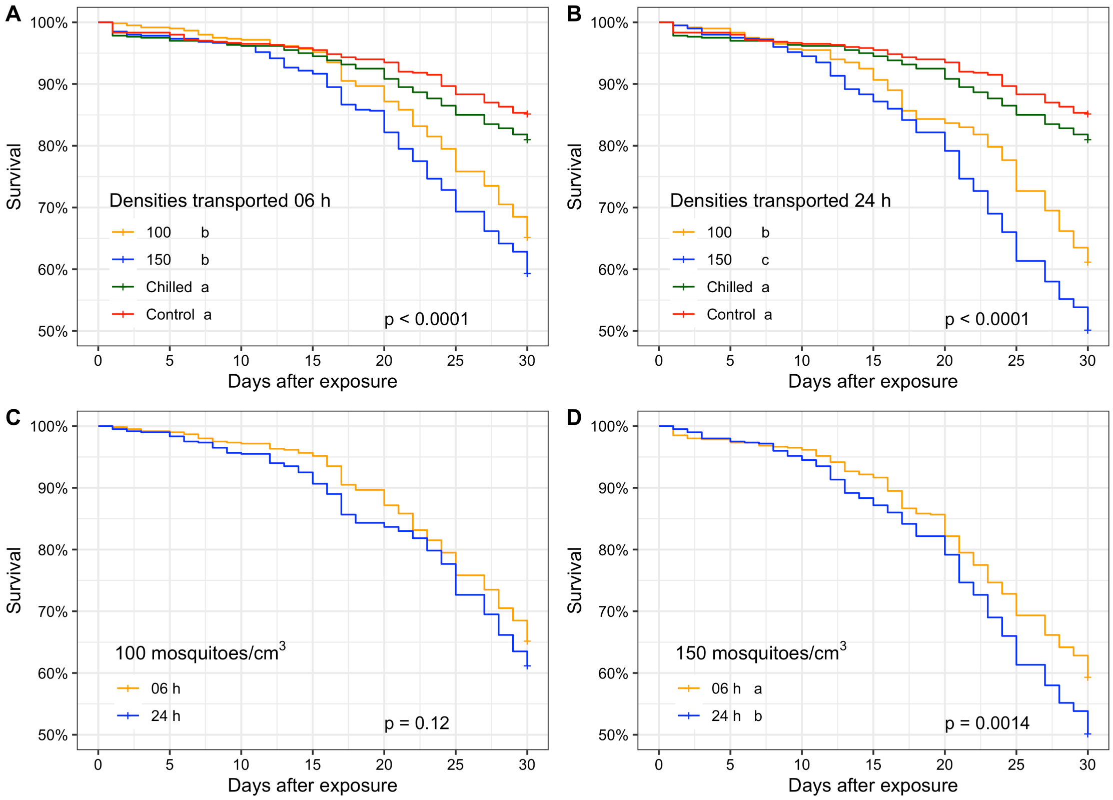

Figure 3. Survival curve for transportation time interval and compaction levels

Insemination

```
## # A tibble: 6 × 2
##   interact   m_percentage
##   <fct>             <dbl>
## 1 control.6h         0.66
## 2 chilled.6h         0.59
## 3 100.6h             0.58
## 4 150.6h             0.49
## 5 100.24h            0.5
## 6 150.24h            0.4
```

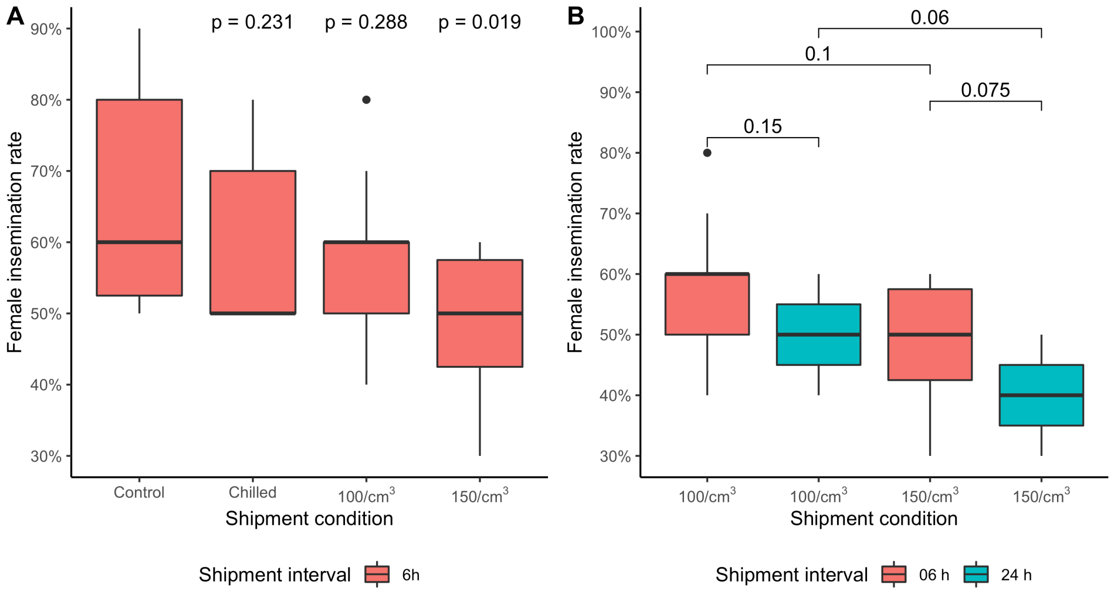

Figure 4. Survival curve for transportation time interval and compaction levels

Spermatheca replenishment level

```
##
##  Wilcoxon rank sum test with continuity correction
##
## data:  p_percent by treatment
## W = 36, p-value = 0.2884
## alternative hypothesis: true location shift is not equal to 0

##
##  Wilcoxon rank sum test with continuity correction
##
## data:  p_percent by treatment
## W = 19.5, p-value = 0.01856
## alternative hypothesis: true location shift is not equal to 0

##
##  Wilcoxon rank sum test with continuity correction
##
## data:  p_percent by time
## W = 49.5, p-value = 0.1519
## alternative hypothesis: true location shift is not equal to 0

##
##  Wilcoxon rank sum test with continuity correction
##
## data:  p_percent by time
## W = 53, p-value = 0.07543
## alternative hypothesis: true location shift is not equal to 0
```

```
##
##  Wilcoxon rank sum test with continuity correction
##
## data:  p_percent by treatment2
## W = 453.5, p-value = 0.964
## alternative hypothesis: true location shift is not equal to 0
```

```
##
##  Wilcoxon rank sum test with continuity correction
##
## data:  p_percent by treatment2
## W = 209.5, p-value = 0.02086
## alternative hypothesis: true location shift is not equal to 0
```

```
##
##  Kruskal-Wallis rank sum test
##
## data:  p_percent by treatment
## Kruskal-Wallis chi-squared = 7.0029, df = 3, p-value = 0.0718
```

```
## # A tibble: 12 × 4
## # Groups:   treatment, time [4]
##    treatment time  insemination_status  mean
##    <fct>     <ord> <chr>               <int>
##  1 100       6h    full                   10
##  2 100       6h    non-inseminated        10
##  3 100       6h    partial                10
##  4 100       24h   full                    7
##  5 100       24h   non-inseminated         7
##  6 100       24h   partial                 7
##  7 150       6h    full                   10
##  8 150       6h    non-inseminated        10
##  9 150       6h    partial                10
## 10 150       24h   full                    7
## 11 150       24h   non-inseminated         7
## 12 150       24h   partial                 7
```

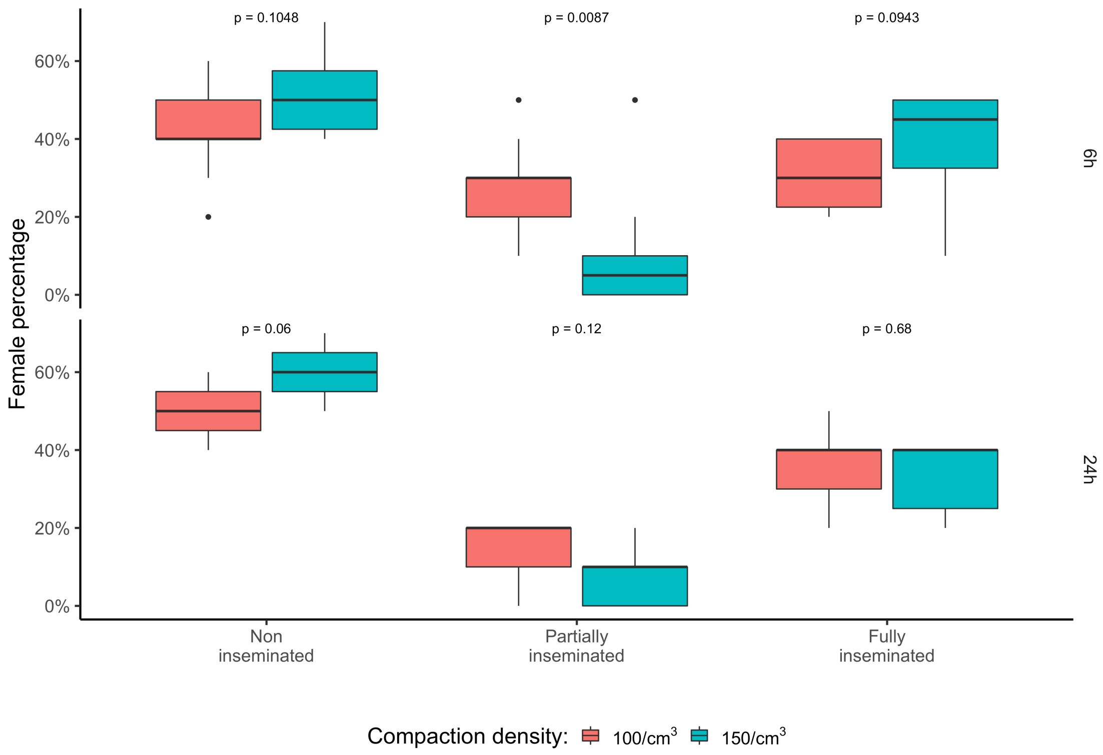

Figure 5. Spermatheca replenishment level of females mated with transported simulated males at different compaction levels

Male escaping rate

```
##
##  Kruskal-Wallis rank sum test
##
## data:  escaping_rate by interact
## Kruskal-Wallis chi-squared = 1.4152, df = 2, p-value = 0.4928

##
##  Kruskal-Wallis rank sum test
##
## data:  escaping_rate by interact
## Kruskal-Wallis chi-squared = 12.292, df = 2, p-value = 0.002142
```

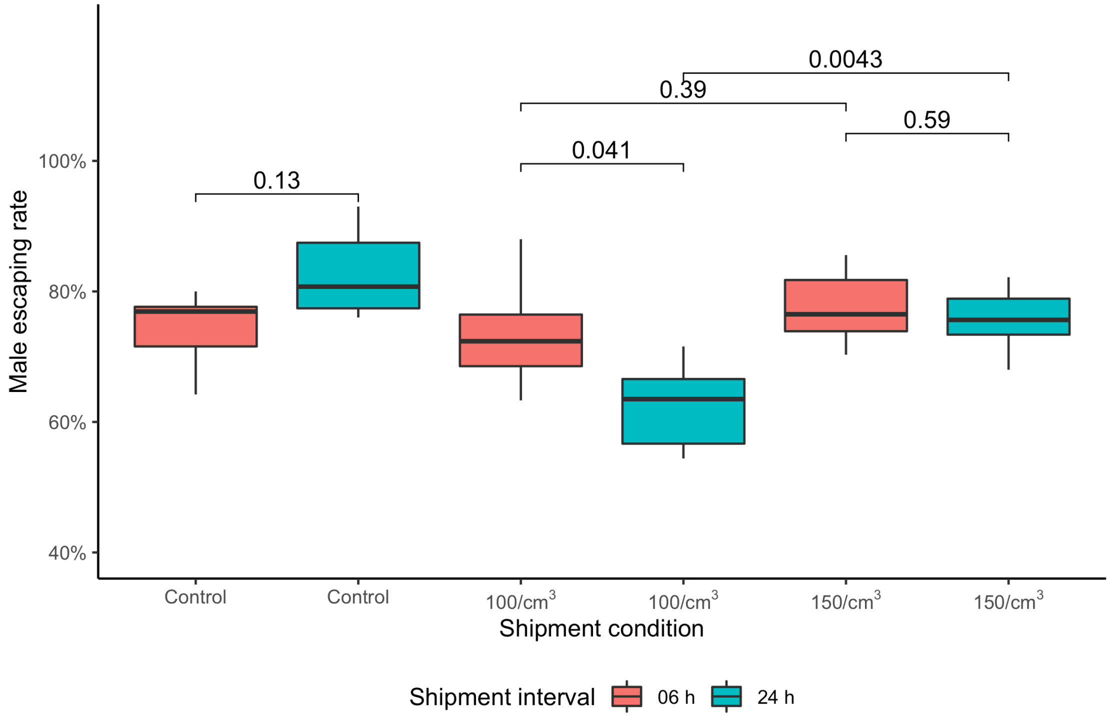

Figure 6. Male ecaping rate of different transportation time intervals (6 and 24h) and at different compaction levels

Physical damage

```
## # A tibble: 4 × 3
## # Groups:   compaction_interval [2]
##   compaction_interval compaction_rate  mean
##   <ord>               <chr>           <dbl>
## 1 6hrs                100/cm3         0.950
## 2 6hrs                150/cm3         0.991
## 3 24hrs               100/cm3         0.933
## 4 24hrs               150/cm3         0.992

##
##  Wilcoxon rank sum test with continuity correction
##
## data:  percentage by interact
## W = 12.5, p-value = 0.01518
## alternative hypothesis: true location shift is not equal to 0

##
##  Wilcoxon rank sum test with continuity correction
##
## data:  percentage by interact
## W = 12, p-value = 0.01282
## alternative hypothesis: true location shift is not equal to 0
```

```
##
##  Wilcoxon rank sum test with continuity correction
##
## data:  percentage by interact
## W = 38.5, p-value = 0.9291
## alternative hypothesis: true location shift is not equal to 0

##
##  Wilcoxon rank sum exact test
##
## data:  percentage by interact
## W = 35, p-value = 0.6965
## alternative hypothesis: true location shift is not equal to 0

##
##  Wilcoxon rank sum exact test
##
## data:  percentage by interact
## W = 50, p-value = 0.4082
## alternative hypothesis: true location shift is not equal to 0

##
##  Wilcoxon rank sum exact test
##
## data:  percentage by interact
## W = 46, p-value = 0.6334
## alternative hypothesis: true location shift is not equal to 0

##
##  Wilcoxon rank sum test with continuity correction
##
## data:  percentage by interact
## W = 49.5, p-value = 0.4229
## alternative hypothesis: true location shift is not equal to 0

##
##  Wilcoxon rank sum test with continuity correction
##
## data:  percentage by interact
## W = 38, p-value = 0.8929
## alternative hypothesis: true location shift is not equal to 0

##
##  Wilcoxon rank sum test with continuity correction
##
## data:  percentage by interact
## W = 10, p-value = 0.008729
## alternative hypothesis: true location shift is not equal to 0

##
##  Wilcoxon rank sum test with continuity correction
```

```
##
## data:  percentage by interact
## W = 16, p-value = 0.03487
## alternative hypothesis: true location shift is not equal to 0
```

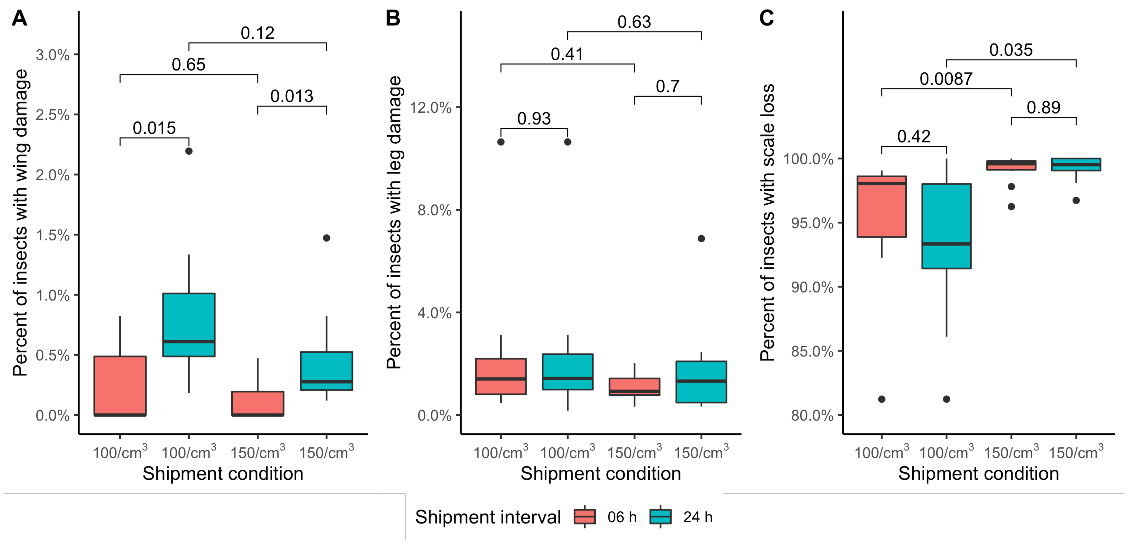

Figure 7. Physical damage analysis considering different body parameters in relation to levels of compaction and transportation time interval.

Supplementary Material

Figure S1

```
##        mean      sd
## 1 1.082895 1.21211

## # A tibble: 8 × 4
## # Groups:   treatment [4]
##   treatment time   mean    sd
##   <chr>     <chr> <dbl> <dbl>
## 1 100       24h    0.81 0.393
## 2 100       6h     1.4  0.885
## 3 150       24h    2.28 0.415
## 4 150       6h     2.48 1.06
## 5 chilled   24h    1    1.41
## 6 chilled   6h     1    1.41
## 7 control   24h    0    0
## 8 control   6h     0    0

##
##  Kruskal-Wallis rank sum test
##
## data:  mortality by interact2
## Kruskal-Wallis chi-squared = 46.239, df = 7, p-value = 7.856e-08
```

```
##
##  Kruskal-Wallis rank sum test
##
## data:  mortality by interact2
## Kruskal-Wallis chi-squared = 23.299, df = 11, p-value = 0.01604
```

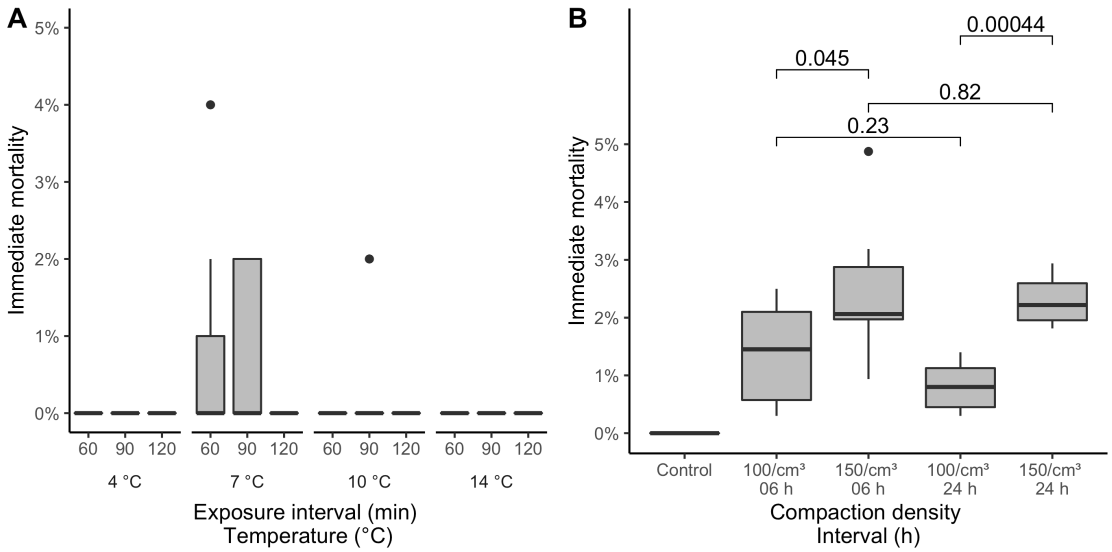

Figure S1. Survival curve for each temperature and time interval that males were submitted

Figure S2

```
## Call:
## survdiff(formula = Surv(time, status) ~ treatment + temperatura,
##     data = handling_data)
##
##                                     N Observed Expected (O-E)^2/E (O-E)^2/
V
## treatment=120, temperatura=4      350       89     68.2  6.341317  6.93787
8
## treatment=120, temperatura=7      350       76     71.2  0.324058  0.35583
8
## treatment=120, temperatura=10     350       69     69.1  0.000169  0.00018
6
## treatment=120, temperatura=14     350       64     71.3  0.746932  0.82033
3
## treatment=60, temperatura=4       350       82     69.1  2.411796  2.64157
6
## treatment=60, temperatura=7       350       72     70.7  0.023395  0.02567
4
## treatment=60, temperatura=10      350       67     69.9  0.123215  0.13509
2
## treatment=60, temperatura=14      350       79     70.5  1.012946  1.11138
7
## treatment=90, temperatura=4       350       64     71.5  0.781165  0.85806
5
```

```
## treatment=90, temperatura=7       350        66       71.7  0.458437  0.50376
4
## treatment=90, temperatura=10      350        64       70.2  0.548802  0.60194
3
## treatment=90, temperatura=14      350        65       70.9  0.493813  0.54207
3
## treatment=Control, temperatura=4  350        58       70.6  2.240701  2.45880
8
##
##  Chisq= 15.7  on 12 degrees of freedom, p= 0.2
```

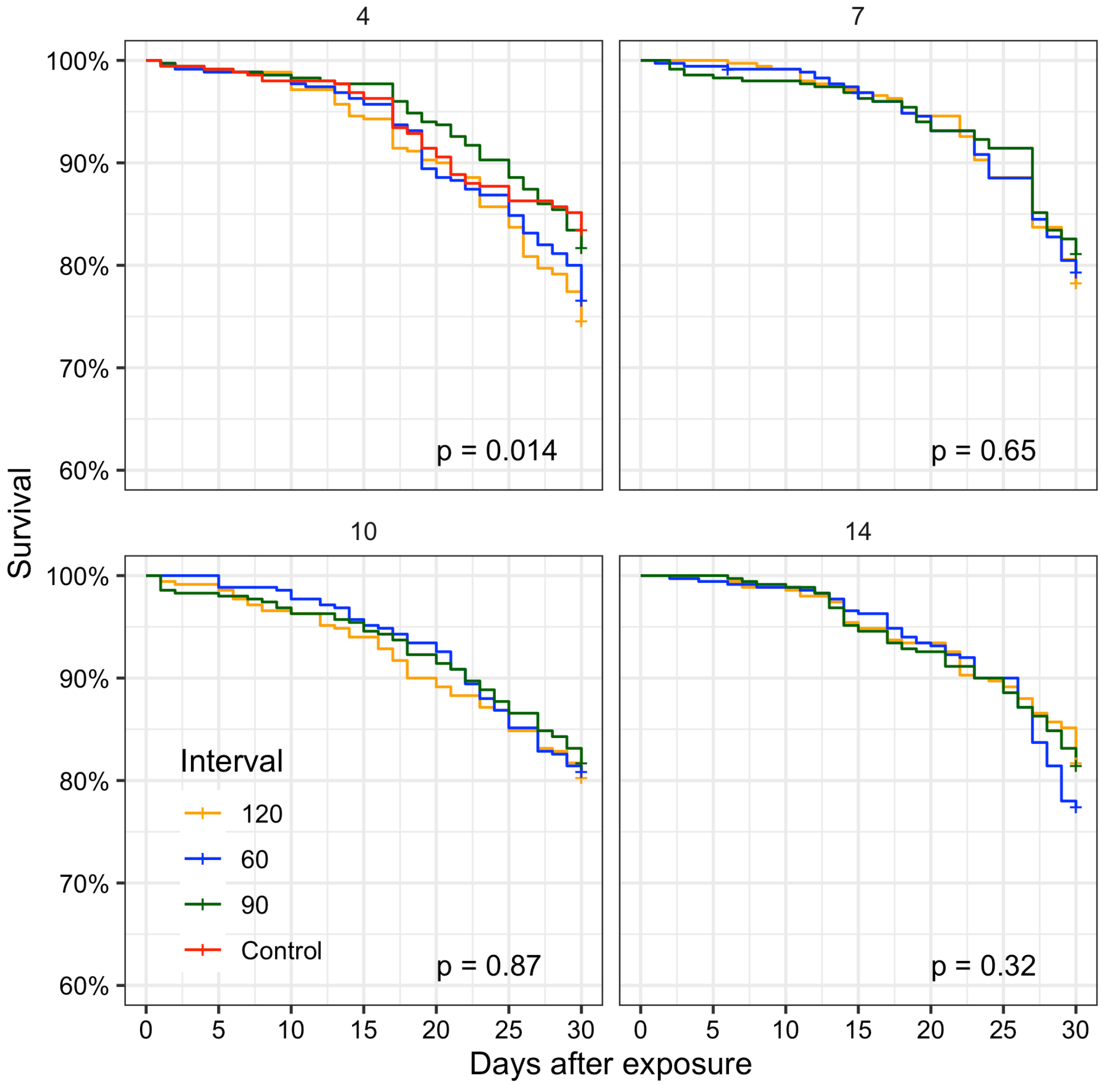

Figure S2. Survival curve for each temperature and time interval that males were submitted

Session information

```
## R version 4.2.0 (2022-04-22)
## Platform: x86_64-apple-darwin17.0 (64-bit)
```

```
## Running under: macOS Big Sur/Monterey 10.16
##
## Matrix products: default
## BLAS:   /Library/Frameworks/R.framework/Versions/4.2/Resources/lib/libRbla
s.0.dylib
## LAPACK: /Library/Frameworks/R.framework/Versions/4.2/Resources/lib/libRlap
ack.dylib
##
## locale:
## [1] en_US.UTF-8/en_US.UTF-8/en_US.UTF-8/C/en_US.UTF-8/en_US.UTF-8
##
## attached base packages:
## [1] stats     graphics  grDevices utils     datasets  methods   base
##
## other attached packages:
##  [1] multcomp_1.4-19   TH.data_1.1-1     MASS_7.3-56       mvtnorm_1.1-3
##  [5] magrittr_2.0.3    rcompanion_2.4.15 survminer_0.4.9   ggpubr_0.4.0
##  [9] survival_3.3-1    forcats_0.5.1     stringr_1.4.0     dplyr_1.0.9
## [13] purrr_0.3.4       readr_2.1.2       tidyr_1.2.0       tibble_3.1.7
## [17] ggplot2_3.3.6     tidyverse_1.3.1
##
## loaded via a namespace (and not attached):
##  [1] matrixStats_0.62.0 fs_1.5.2           lubridate_1.8.0    httr_1.4.2
##  [5] tools_4.2.0        backports_1.4.1    utf8_1.2.2         R6_2.5.1
##  [9] nortest_1.0-4      DBI_1.1.2          colorspace_2.0-3   withr_2.5.0
## [13] tidyselect_1.1.2   gridExtra_2.3      Exact_3.1          compiler_4.2
.0
## [17] cli_3.3.0          rvest_1.0.2        expm_0.999-6       xml2_1.3.3
## [21] sandwich_3.0-1     labeling_0.4.2     scales_1.2.0       lmtest_0.9-4
0
## [25] survMisc_0.5.6     proxy_0.4-26       multcompView_0.1-8 digest_0.6.2
9
## [29] rmarkdown_2.14     pkgconfig_2.0.3    htmltools_0.5.2    dbplyr_2.1.1
## [33] fastmap_1.1.0      rlang_1.0.2        readxl_1.4.0       rstudioapi_0
.13
## [37] farver_2.1.0       generics_0.1.2     zoo_1.8-9          jsonlite_1.8
.0
## [41] car_3.0-12         modeltools_0.2-23  Matrix_1.4-1       Rcpp_1.0.8.3
## [45] DescTools_0.99.45  munsell_0.5.0      fansi_1.0.3        abind_1.4-5
## [49] lifecycle_1.0.1    stringi_1.7.6      yaml_2.3.5         carData_3.0-
5
## [53] rootSolve_1.8.2.3  plyr_1.8.7         grid_4.2.0         parallel_4.2
.0
## [57] crayon_1.5.1       lmom_2.8           lattice_0.20-45    cowplot_1.1.
1
## [61] haven_2.5.0        splines_4.2.0      hms_1.1.1          knitr_1.38
## [65] pillar_1.7.0       boot_1.3-28        gld_2.6.4          ggsignif_0.6
.3
## [69] codetools_0.2-18   stats4_4.2.0       reprex_2.0.1       glue_1.6.2
## [73] evaluate_0.15      data.table_1.14.2  modelr_0.1.8       vctrs_0.4.1
```

```
## [77] tzdb_0.3.0        cellranger_1.1.0   gtable_0.3.0       km.ci_0.5-6
## [81] assertthat_0.2.1   xfun_0.30          coin_1.4-2         libcoin_1.0-
9
## [85] xtable_1.8-4       broom_0.8.0        e1071_1.7-9        rstatix_0.7.
0
## [89] class_7.3-20       KMsurv_0.1-5       ellipsis_0.3.2
```
