# Supplementary figures and images for: Exploring Conditions for Handling Packing and Shipping Aedes aegypti Males to Support an SIT Field Project in Brazil
Source: Insects. 2022 Sep 25;13(10):871. doi: 10.3390/insects13100871 (PMC9604236; doi:10.3390/insects13100871)

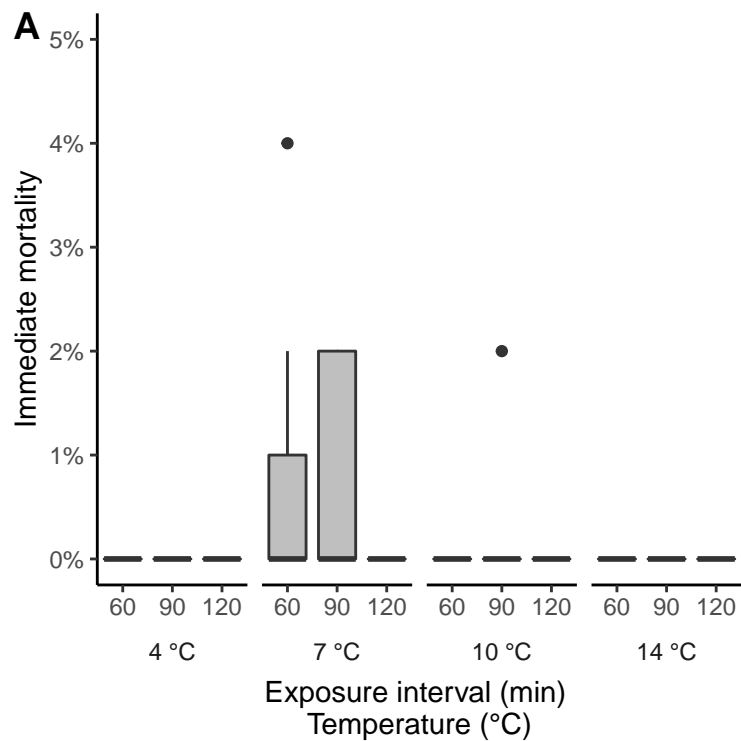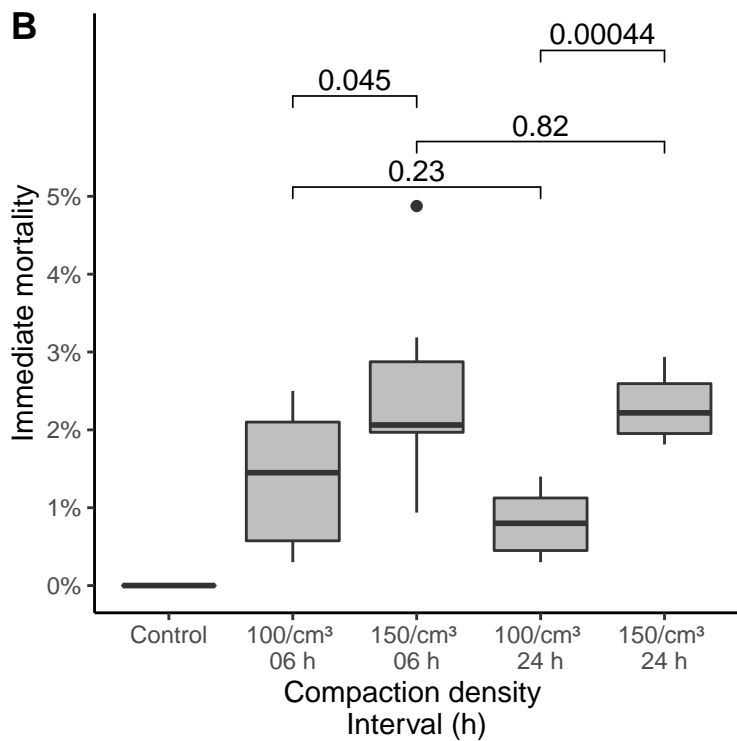

Supplement: Supplementary file 1 [file insects-13-00871-s001.zip › figure_FS1.pdf]

4

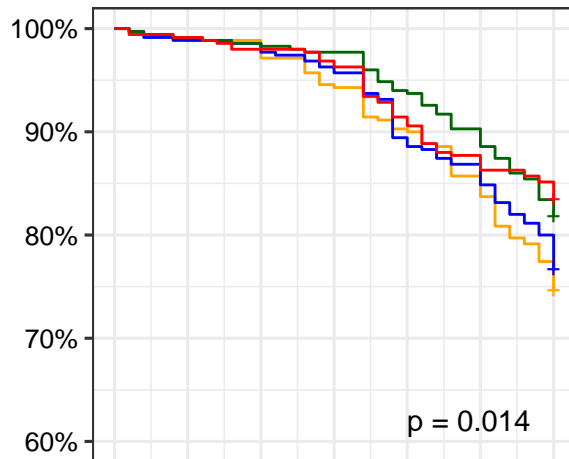

7

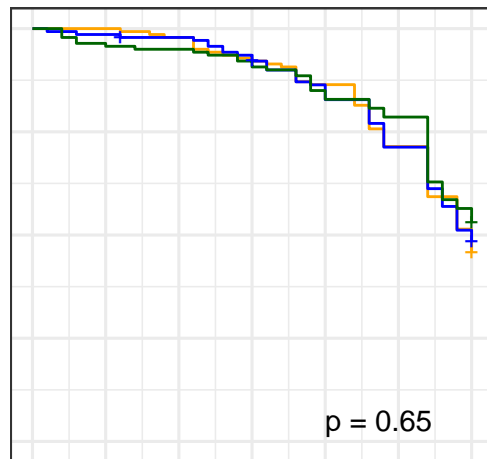

10

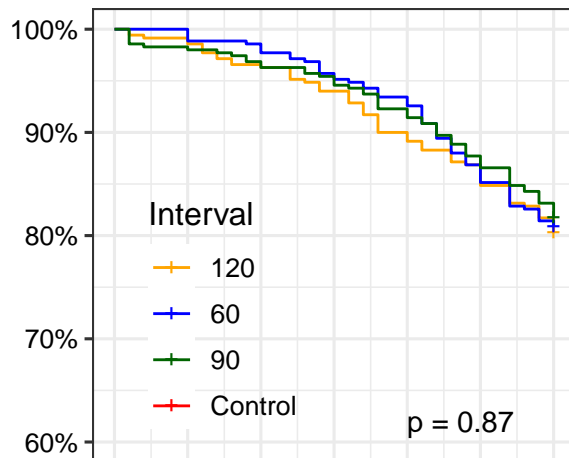

14

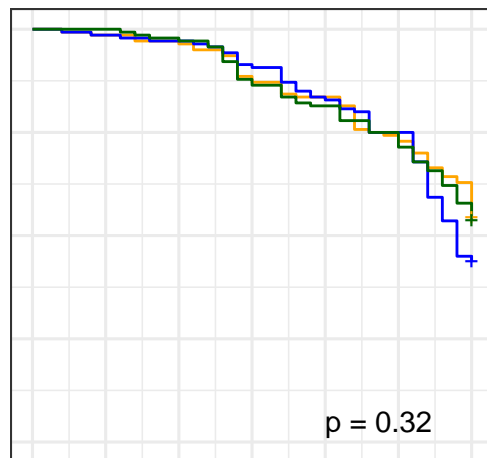

Days after exposure

Supplement: Supplementary file 1 [file insects-13-00871-s001.zip › figure_FS2.pdf]
